# Impact of unmet health-related need on suicidal behavior in Korean adults: a retrospective nationwide cohort study

**DOI:** 10.1038/s41598-024-63200-x

**Published:** 2024-06-11

**Authors:** Youn Huh, Ju Young Huh, Yerim Jeon, Jun Hyung Lee

**Affiliations:** 1 Department of Family Medicine, Uijeongbu Eulji Medical Center, Eulji University, Seongnam-si, Gyeonggi-do Republic of Korea; 2 Institute of Knowledge and Culture, Duksung Women’s University, Seoul, Republic of Korea; 3 Department of Neurosurgery, Asan Medical Center, Seoul, Republic of Korea; 4 Department of Family Medicine, Inje University Ilsan Paik Hospital, College of Medicine, Inje University, 170, Juhwa-ro, Ilsanseo-gu, Goyang-si, Seoul, Gyeonggi-do 10380 Republic of Korea

**Keywords:** Suicidal plans, Suicidal attempt, Suicidal behavior, Unmet health-related need, Korean, Adult, Health care, Risk factors

## Abstract

We aimed to evaluate the association of unmet health-related need with suicidal behaviors among Korean adults. We included 26,219 adults (13,937 men and 17,788 women) aged ≥ 19 years from the Korea National Health and Nutrition Examination Survey (2015–2020). Suicidal behavior included suicidal plan and attempt. We analyzed the odds ratios and 95% confidence intervals of suicidal behaviors according to unmet health-related need via multivariable logistic regression analysis and performed stratified analyses according to sex, age, income, education, and type of insurance. Of the participants, 9.6% had unmet health-related need. Suicidal plans and attempts had 1.3% and 0.5% of the participants, respectively. The prevalence of suicidal plans and attempts was 0.9% and 0.4% among participants without unmet health-related need and 3.1% and 1.0% among those with such need, respectively. The odd ratios of suicidal plans and attempts increased significantly among participants with unmet health-related need compared to those without. In subgroup analysis, most subgroups showed similar results, except for suicidal plan and attempt in the 45–64 age group, high education, and medical care and suicidal attempt in 19–44 age group, low-income, and unmarried. Unmet health-related need was independently associated with suicidal plan and attempt. A policy alternative is needed for these associations.

## Introduction

Approximately 700,000 people die by suicide each year worldwide^1^. In the US, suicide was the 12th leading cause of death, and the second to fifth cause of death among young people in 2020^2^. In addition, from 2000 to 2020, the prevalence of suicide tended to increase steadily^2^. Among Organization for Economic Co-operation and Development (OECD) countries, South Korea has a high prevalence of suicide, and its annual suicide prevalence is approximately twice the average of that of OECD countries^3^. Previous studies have shown that that suicide occurs in progressive stages in the following order: suicidal ideation, suicidal plan, and suicidal attempt^4,5^. In addition, previous suicidal attempt is a key risk factor for suicide in people of all ages^1^. Therefore, it is important to manage and prevent suicidal plans and attempts in terms of medical policy.

Unmet health-related need is defined as a subjective evaluation of healthcare need that are not adequately fulfilled^6^. The incidence of unmet health-related need is higher among women, those with lower income and education level, and those with a lack of health insurance^7,8^. Unmet health-related need is associated with an increased risk of severe disease, complication, decreased quality of life, and death^9,10^. Unmet health-related need has been associated with psychologic symptoms, such as depression among older adults^11,12^ and anxiety among cancer patients^13^, as unmet health-related need leads to worse health and disability.

Depression is one of the important risk factors of suicide. However, few studies have evaluated representative nationwide data, and evidence of an association of unmet health-related need and suicidal plan and attempt among adults is limited. Therefore, the purpose is this study evaluated the impact of unmet health-related needs on suicidal plans and attempts among Korean adults aged ≥ 19 years using nationwide cohort data from the Korea National Health and Nutrition Examination Survey (KNHANES). In addition, we conducted stratified analyses according to sex, age, income, education, and type of insurance.

## Methods

### Data source and study population

We used data from the KNHANES between 2015 and 2020. The KNHANES includes a stratified, multistage probability sampling of the household units that participated in the survey. It provides data on demographic characteristics, health behaviors, and health status obtained from personal interviews as well as data from physical examinations performed in mobile examination centers, including anthropometric measurements and blood samples. Our study sample initially included 38,073 KNHANES participants aged ≥ 19 years between 2015 and 2020. We excluded 11,854 participants with missing data for any of the variables. Therefore, our study sample included 26,219 adults aged ≥ 19 years (11,664 men and 14,555 women).

## Ethics approval

The present study was approved by the Institutional Review Board of the Korea Center for Disease Control and Prevention (IRB No: 2018-01-03-P-A, 2018-01-03-C-A, and 2018-01-03-2C-A). The requirement for informed consent was waived because of anonymized and de-identified database. In addition, the present study was performed in accordance with the Declaration of Helsinki.

### Study outcomes

In the of questionnaire section on suicidal behaviors of the KNHANES, participants were asked to answer the following question: “Have you seriously planed suicide in the last year?” and “Have you seriously attempted suicide in the last year?” Individuals who answered this question with “yes” were considered to have made a suicidal plan or suicidal attempt.

### Definitions of unmet health-related need

In the KNHANES, the following question was asked to the participants regarding medical use: “During the past year, have you ever needed to use a medical care (examination or treatment) at a hospital or clinic (excluding dentistry) but were not able to receive it?” Individuals who answered this question with “yes” were considered to have a unmet health-related need.

### Covariates

Household income levels were divided into first quartile groups and others. Education status was classified based on 12 years. Smoking status and alcohol consumption were dichotomized according to a history of smoking or alcohol consumption. Regular physical activity was defined as moderate-intensity physical activity for more than 150 min per week. Types of health insurance were classified into medical care and others. According to marital status, participants were divided into married and unmarried. The participants were asked the following question regarding subjective health status: “How do you usually think about your health status?” Individuals who answered this question with “bad” and “very bad” were considered to have poor subjective health status. Comorbidities included hypertension, diabetes mellitus, dyslipidemia, and depressive disorder, which were determined by physical diagnosis. Anthropometric parameters included height, body weight, and waist circumference (WC), which were measured by trained staff using standard protocols and equipment. The body mass index (BMI) was calculated by dividing body weight by height^2^ (kg/m^2^). According to the definition of obesity by the World Health Organization for the Asia Pacific Region^14^, BMI was classified as < 25.0 and ≥ 25.0 kg/m^2^. Three blood pressure (BP) measurements were obtained using a sphygmomanometer with the participant in a sitting position, and the averages of the second and third measurements were used in analyses. A blood sample including total cholesterol and fasting plasma glucose was collected from each participant after an overnight 8-h fast.

### Statistical analyses

We combined data from the 2015–2020 KNHANES based on its raw data analysis guidelines. Following the complex sample design, we conducted all analyses by assigning dispersed stratification estimates, stratification variables, and weighted sample values. We analyzed continuous variables using a general linear model and presented them as means and standard errors. We presented categorical variables as ratios and standard errors and analyzed them using the chi-square test. Furthermore, to determine the association between unmet health-related need as the independent variable and suicidal plan and attempt as the dependent variable, we conducted multivariable logistic regression analysis and calculated the odds ratios (ORs) and 95% confidence intervals (CIs). We used five models: Model 1 was not adjusted; Model 2 was adjusted for age, sex, and marital status; Model 3 was additionally adjusted for education, household income, and type of health insurance; Model 4 was additionally adjusted for obesity, cigarette smoking, alcohol drinking, and regular physical activity; and Model 5 was additionally adjusted for subjective health status, depressive disorder, and comorbidities (hypertension, diabetes mellitus, and dyslipidemia). In addition, we performed stratified analyses according to sex, age, income, education, and type of insurance and set statistical significance at *p* < 0.05. All analyses were performed using SPSS software (version 24.0; IBM Corp., Armonk, NY, USA).

## Results

### Basic characteristics according to the presence of unmet health-related need

Table 1 presents the basic characteristics of 26,219 adults aged ≥ 19 years. The prevalence of men was higher among participants without unmet health-related need. The mean age was 45.3 ± 0.2 years. The mean values of height, weight, WC, and diastolic BP were higher among participants without unmet health-related need. The proportions of low income, low education, current smokers, medical care, and depressive disorder were higher among participants with unmet health-related need. The proportion of patients with dyslipidemia was higher among participants with unmet health-related need. The prevalence of alcohol drinkers and poor subjective health status was higher among participants without unmet health-related need.

**Table 1:** Basic characteristics according to the presence of unmet health-related need.

	Unmet health-related need		*p* value*
	(+)	(−)	
	N = 2303	N = 13,028	
N	2303	23,916	
Sex (male)	39.4 (1.2)	50.8 (0.3)	< 0.001
Age (years)	45.3 ± 0.4	45.4 ± 0.2	0.673
Height (cm)	163.8 ± 0.2	165.5 ± 0.1	< 0.001
Weight (kg)	64.9 ± 0.4	66.0 ± 0.1	0.003
Waist circumference (cm)	82.2 ± 0.3	82.7 ± 0.1	0.048
BMI (kg/m^2^)	24.0 ± 0.1	24.0 ± 0.0	0.554
Systolic BP (mmHg)	116.0 ± 0.4	116.4 ± 0.1	0.286
Diastolic BP (mmHg)	74.7 ± 0.3	75.8 ± 0.1	< 0.001
FPG (mg/dL)	99.3 ± 0.7	99.1 ± 0.2	0.783
Total cholesterol (mg/dL)	193.0 ± 0.9	192.2 ± 0.3	0.390
Income (lowest quartile)	18.7 (0.9)	12.2 (0.4)	< 0.001
Education (< 13 years)	60.2 (1.3)	55.0 (0.6)	< 0.001
Alcohol drinker	58.8 (1.2)	61.4 (0.4)	0.036
Current smoker	22.7 (1.1)	19.8 (0.4)	< 0.001
Regular physical activity	48.4 (1.3)	48.1 (0.4)	0.800
Medical care	5.0 (0.6)	2.4 (0.1)	< 0.001
Unmarried	27.3 (1.2)	25.6 (0.5)	0.161
Bad subjective health status	45.8 (1.3)	48.9 (0.4)	0.018
Hypertension	16.9 (0.9)	17.3 (0.3)	0.671
Diabetes mellitus	6.1 (0.5)	6.8 (0.2)	0.237
Dyslipidemia	16.6 (0.9)	14.6 (0.3)	0.028
Depressive mood (+)	6.8 (0.6)	3.6 (0.1)	< 0.001

BMI, body mass index; BP, blood pressure; FPG, fasting plasma glucose.

Values are presented as mean ± standard error or percentage (standard error).

*p values were obtained using the chi-squared test.

### The prevalence of suicidal behavior according to the presence of unmet health-related need

Table 2 shows the prevalence of suicidal plans and attempts according to the presence of unmet health-related need. The proportions of suicidal plan and attempt were 0.9% and 0.4% in participants without unmet health-related need, and 3.1% and 1.0% in those with unmet health-related need, respectively (*p* < 0.001). In men, the prevalence of suicidal plan and attempt were 0.8% and 0.3% in participants without unmet health-related need, and 3.6% and 0.9% in those with unmet health-related need, respectively (*p* < 0.001 in suicidal plan and *p* < 0.002 in suicidal attempt). In women, the proportions of suicidal plan and attempt were 1.1% and 0.5% in participants without unmet health-related need, and 2.7% and 1.1% in those with unmet health-related need, respectively (*p* < 0.001 in suicidal plan and *p* < 0.003 in suicidal attempt).

**Table 2:** The prevalence of suicidal behavior according to the presence of unmet health-related need.

		Suicidal plan (N = 337)		*p*-value*	Suicidal attempt (N = 121)		*p* value*
		Unmet health-related need			Health-related unmet usage		
		(+)	(−)		(+)	(−)	
	N	80	257		26	95	
Total		3.1 (0.4)	0.9 (0.1)	< 0.001	1.0 (0.2)	0.4 (0.0)	< 0.001
Sex	Men	3.6 (0.8)	0.8 (0.1)	< 0.001	0.9 (0.4)	0.3 (0.1)	0.002
	Women	2.7 (0.4)	1.1 (0.1)	< 0.001	1.1 (0.3)	0.5 (0.1)	0.003

Values are presented as percentage (standard error).

*p values were obtained using the chi-squared test.

### Association between unmet health-related need and suicidal behavior

Table 3 presents the ORs of suicidal plans and attempt based on the unmet health-related need. After adjusting for confounding variables (model 5), compared to participants without unmet health-related need, the OR for suicidal plan was 2.44 times higher in those with unmet health-related need (OR 2.44, 95% CI 1.73–3.45). The OR for suicidal attempt increased by 88% among participants with unmet health-related need (OR 1.88, 95% CI 1.09–3.25), compared to among those without unmet health-related need.

**Table 3:** Odds ratio (ORs) and 95% confidence interval (CIs) for suicidal behavior according to the presence of unmet health-related need.

	Unmet health-related need	Model 1	Model 2	Model 3	Model 4	Model 5
		OR (95% CI)	OR (95% CI)	OR (95% CI)	OR (95% CI)	OR (95% CI)
Suicidal plan	(−)	1 (ref.)	1 (ref.)	1 (ref.)	1 (ref.)	1 (ref.)
	(+)	3.35 (2.49–4.50)	3.22 (2.37–4.37)	2.83 (2.06–3.89)	2.74 (2.00–3.76)	2.44 (1.73–3.45)
Suicidal attempt	(−)	1 (ref.)	1 (ref.)	1 (ref.)	1 (ref.)	1 (ref.)
	(+)	2.89 (1.83–4.56)	2.67 (1.68–4.24)	2.41 (1.50–3.88)	2.24 (1.39–3.61)	1.88 (1.09–3.25)

OR, odds ratio; CI, confidence interval.

Values were calculated by multivariable logistic regression analysis.

Model 1 was not adjusted.

Model 2 was adjusted for age, sex, and marital status.

Model 3 was adjusted for age, sex, marital status, education, household income, and type of health insurance.

Model 4 was adjusted for age, sex, marital status, education, household income, type of health insurance, obesity, cigarette smoking, alcohol drinking, and regular physical activity.

Model 5 was adjusted for age, sex, marital status, education, household income, type of health insurance, obesity, cigarette smoking, alcohol drinking, regular physical activity, subjective health status, depressive disorder, and comorbidities (hypertension, diabetes mellitus, and dyslipidemia).

### Subgroup analyses

Table 4 presents the subgroup analyses of the association between unmet health-related need as the independent variable and suicidal plan and attempt. In subgroup analyses, except for adults aged 45–64 years, those with education ≥ 13 years, and those with medical care, the ORs for suicidal plan increased from 1.74 (OR 1.74, 95% CI 1.17–2.60 in women) to 4.41 (OR 4.41, 95% CI 2.50–7.76 in men) times in participants with unmet health-related need compared to those without unmet health-related need. Compared with those with unmet health-related need, the OR for suicidal attempt increased among individuals with unmet health-related need among those aged ≥ 65 years, without low income, with education < 13 years, married, and without medical care.

**Table 4:** Subgroup analyses.

	Suicidal plan		P for interaction	Suicidal attempt		P for interaction
	Unmet health-related need			Unmet health-related need		
	(−)	(+)		(−)	(+)	
	OR (95% CI)			OR (95% CI)		
Sex			0.013			0.331
Men	1 (ref.)	**4.41 (2.50–7.76)**		1 (ref.)	2.85 (0.97**–**8.35)	
Women	1 (ref.)	**1.74 (1.17–2.60)**		1 (ref.)	1.74 (0.92**–**3.28)	
Age (years)			0.014			0.090
19–44	1 (ref.)	**3.02 (1.67–5.46)**		1 (ref.)	2.07 (0.92**–**4.68)	
45–64	1 (ref.)	1.31 (0.67**–**2.56)		1 (ref.)	1.08 (0.42**–**2.80)	
≥ 65	1 (ref.)	**4.04 (2.37–6.90)**		1 (ref.)	**3.53 (1.67–7.47)**	
Household income			0.308			0.462
Lowest	1 (ref.)	**2.11 (1.25–3.56)**		1 (ref.)	1.61 (0.64**–**4.05)	
Others	1 (ref.)	**2.70 (1.75–4.16)**		1 (ref.)	**2.12 (1.08–4.16)**	
Education (years)			0.510			0.969
< 13	1 (ref.)	**2.67 (1.81–3.93)**		1 (ref.)	**1.93 (1.04–3.59)**	
≥ 13	1 (ref.)	1.54 (0.70**–**3.40)		1 (ref.)	2.15 (0.61**–**7.63)	
Marital status			0.333			0.470
No married	1 (ref.)	**3.36 (1.74–6.50)**		1 (ref.)	1.89 (0.65**–**5.49)	
Married	1 (ref.)	**2.26 (1.53–3.35)**		1 (ref.)	**2.16 (1.17–3.98)**	
Type of health insurance			0.165			0.068
Medical care	1 (ref.)	1.31 (0.58**–**2.95)		1 (ref.)	0.39 (0.07**–**2.07)	
Others	1 (ref.)	**2.67 (1.86–3.85)**		1 (ref.)	**2.31 (1.35–3.95)**	

OR, odds ratio; CI, confidence interval.

Values are presented as odds ratios (95% confidential intervals) using multivariable logistic regression analysis after adjusting for age, sex, marital status, education, household income, type of health insurance, obesity, cigarette smoking, alcohol drinking, regular physical activity, subjective health status, depressive disorder, and comorbidities (hypertension, diabetes mellitus, and dyslipidemia).

Significant values are in bold.

## Discussion

In this study, we investigated the association of unmet health-related need with suicidal behaviors (suicidal plan and attempt) among Korean adults aged ≥ 19. The present study showed that the ORs for suicidal plan and attempt significantly increased among individuals with unmet health-related need compared with those with unmet health-related need. These associations were remained consistent even after stratifying by sex, age, household income, education status, marital status, and type of health insurance. In particular, suicidal plan should be managed among men and older people because they are more prominent groups.

A recent study including 1202 Belgians aged 15–80 years demonstrated that the prevalence of unmet mental health need increased among individuals with suicidal ideation compared to those without^15^. The proportions of fully and partial unmet need for mental health for suicidal thoughts were 2.3% and 1.3% of total participants, respectively^15^. Another study including 142,322 US adults aged ≥ 19 years showed that suicidal ideation and behavior were associated with an increased prevalence of healthcare utilization^16^. A study of approximately 7500 American pregnant women showed that the OR for suicidal behavior increased 5.64 (OR 5.64, 95% CI 3.55–8.97) times in pregnant women with unmet mental health need compared with those without this during pregnancy^17^. The present study is significant in showing the associations between unmet health-related need—including mental and physical unmet need—and suicidal plan and attempt among the general population using Korean nationally representative data.

Unmet health-related need is important for physical and mental health status as well as social problem^18^. In addition, health-related quality of life was associated with unmet health-related need^19^, and that of Korea was lower than the mean for OECD countries^20^. The causes of unmet health-related need included high out-of-pocket and medical costs and underlying chronic diseases despite Korea’s well-established health insurance system^21^. In Korea, factors associated with unmet health-related need included age, physical illness, income, type of health insurance, occupation, education status, and subjective health status^22,23^. In addition, in another study including Korean adults, unmet health-related need continued to increase from 17% in 2005 to 20.3% in 2010^24^. Therefore, policymakers need to ameliorate unmet health-related need for vulnerable populations.

Furthermore, we showed that these associations mostly remained consistent after various subgroups, highlighting the causal associations of unmet health-related need with suicidal behavior. The ORs for suicidal plan increased among participants with unmet health-related need, except those aged 45–64 years, with ≥ 13 years of education, and with medical care. Interestingly, these associations also remained in the association between unmet health-related need and suicidal attempt among participants aged ≥ 65 years, those without low-income, with < 13 years of education, married, those without medical care. However, people aged 19–64 years, low-income, with ≥ 13 years of education, unmarried, and those with medical care did not show these tendencies. People aged 19–64 years and with high education were likely to having stable employment, sense of belonging, and social support, which were protective factors for suicide or suicidal behavoirs^25^. Individuals with low-income, unmarried, and with medical care excluded these tendencies because they were possible multicollinearity and number of individuals with medical care was small. The association between unmet health-related need and suicidal plan was more prominent in men and participants aged ≥ 65 years. Therefore, this study suggests that alternatives to suicidal plan and attempt are needed for these vulnerable groups.

It is necessary a multifaceted approach to prevent the risk of suicidal behaviors associated with unmet health related need. First of all, it is necessary to identify the vulnerable groups with unmet health related need such as low-income, having chronic diseases, and bad subjective health status^26,27^. In addition, suicide prevention programs implement individuals and socially to prevent suicide in health system, such as the Colorado National Collaborative: connectedness, economic stability and support, education and awareness, access to suicide safer care, lethal means safety, and postvention^28^.

The present study has some limitations. First, we could not establish causal relationships between unmet health-related need and suicidal plan and attempt because the present study was a cross-sectional study. Second, because unmet health-related need was based on the participants’ subjective judgement, objective indicators of the unmet health-related need could not be used. Moreover, regarding the health status and lifestyle of the participants based on self-reported questionnaires, the data might have been subjected to recall bias. Third, the KNHANES included only a questionnaire on unmet health-related need and did not separately include unmet mental and physical health need. However, although the definition of unmet health-related need was insufficient, the associations of unmet health-related need with suicidal behaviors was investigated using large-scale nationwide data. In addition, we considered various confounding variables and performed subgroup analyses. Finally, although we might have considered some of the factors that influenced the study outcomes, all confounding variables might not have been accounted for (e.g., previous suicidal attempt and family history of mental diseases). Despite these limitations, our study demonstrates that unmet health-related need was associated with suicidal plan and attempt as an important social problem among Korean adults aged ≥ 19 years.

In conclusion, using nationally representative data, we showed that unmet health-related need was independently associated with suicidal behaviors, including suicidal plan and attempt. These associations remained consistent in most subgroups. To prevent suicidal behavior, which is a social problem worldwide, it is necessary to reduce unmet health-related need among the general adults aged ≥ 19 years. To reduce these associations, policy alternatives are needed.

## Data Availability

The datasets used and/or analyzed during the current study available from the corresponding author on reasonable request.
